# VarMap: a web tool for mapping genomic coordinates to protein sequence and structure and retrieving protein structural annotations

**DOI:** 10.1093/bioinformatics/btz482

**Published:** 2019-06-13

**Authors:** James D Stephenson, Roman A Laskowski, Andrew Nightingale, Matthew E Hurles, Janet M Thornton

**Affiliations:** 1 European Molecular Biology Laboratory-European Bioinformatics Institute (EMBL-EBI), Hinxton, CB10 1SD, UK; 2 Wellcome Trust Sanger Institute, Wellcome Trust Genome Campus, Hinxton, CB10 1SD, UK

## Abstract

**Motivation:**

Understanding the protein structural context and patterning on proteins of genomic variants can help to separate benign from pathogenic variants and reveal molecular consequences. However, mapping genomic coordinates to protein structures is non-trivial, complicated by alternative splicing and transcript evidence.

**Results:**

Here we present VarMap, a web tool for mapping a list of chromosome coordinates to canonical UniProt sequences and associated protein 3D structures, including validation checks, and annotating them with structural information.

**Availability and implementation:**

https://www.ebi.ac.uk/thornton-srv/databases/VarMap.

**Supplementary information:**

Supplementary data are available at *Bioinformatics* online.

## 1 Introduction

The consequence of variants affecting protein sequence depends on the structural context and chemical environment. Understanding these elements has the potential of both uncovering the biochemical consequences of the change, and of identifying ‘hot spots’ where several variants from different individuals occur within close spatial proximity in the same protein. However, to benefit from the added information 3D protein structures can provide, an accurate mapping between genomic coordinates and the corresponding protein sequence, and structure, is required. Inaccurate mapping may lead to misleading variant interpretation.

**Fig. 1.**
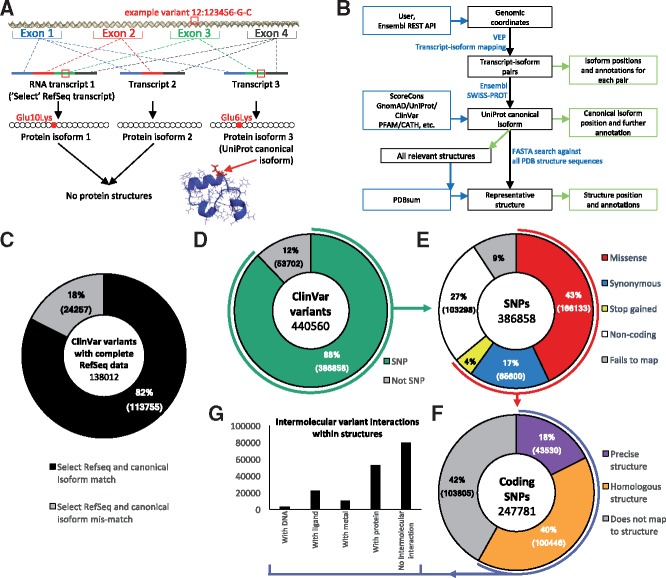
Mapping from genomic coordinates to protein sequence and structure. (**A**) Example missense variant observed on chromosome 12, position 123456, DNA change G/C. Three different transcripts are possible via alternative splicing. Transcript 1 is the longest and is designated as the RefSeq select reference transcript. Three protein isoforms can be created by translating the transcripts. Isoform 3 is designated as the canonical protein isoform in UniProt. The original DNA variant can be mapped onto isoforms 1 and 3, but not to isoform 2 as exon 3 has been spliced out. Isoforms 1 and 2 do not have a corresponding protein 3D structure, whereas isoform 3 does. VarMap maps from the isoform position to the position in the representative structure. (**B**) Simplified schema for mapping from variant genomic coordinates to protein sequence and structure using VarMap. A more detailed version is available in the Supplementary Materials and on the VarMap website. (**C**) Shows the percentages of ClinVar variants belonging to a gene whose translated Select RefSeq transcript is identical to the UniProt canonical isoform sequence (black) and those which do not (grey). ClinVar file used: clinvar_20190211.vcf. (**D**) The percentage of genomic coordinates in ClinVar which are SNPs. (**E**) A breakdown of the SNP variant types. (**F**) The percentage of coding SNPs which can be mapped directly to the exact human structure and those which can be mapped to homologous structures. (**G**) Of the variants which can be mapped to structure, the number which have direct contacts with DNA, metals, ligands and protein as derived from every closely related protein for each variant. The VarMap output from the ClinVar dataset used here is available on the VarMap website. A description of the methods used to generate these plots is available in the Supplementary Material

Alternative splicing makes mapping genomic coordinates to protein sequence non-trivial. As Figure 1A shows, a single coding region can be alternatively spliced into several different transcripts; which of these is expressed may depend on tissue type or developmental stage. Each transcript can result in a different isoform of the same protein. Choosing the relevant transcript is thus a complex matter. In most cases, one of the transcripts is identified as the ‘RefSeq Select transcript’, chosen according to criteria described by NCBI (O'Leary *et al.*, 2016), and has a corresponding protein sequence. Proteins in UniProt also have a reference, or ‘canonical’, sequence (UniProt, 2019). However, as the translated select RefSeq and canonical UniProt sequences are independently derived, they often differ [in 18% of cases in the ClinVar database (Landrum *et al.*, 2018) (Fig. 1C)]—resulting in different numbering of the residues.

## 2 Materials and methods

The user uploads a tab-separated file of genomic coordinates, identifiers (optionally), reference and variant alleles. For files of fewer than 20 coordinates, VarMap runs in real time. For larger files, it runs in batch mode on a processor farm, a link to the results being e-mailed to the user. VarMap performs a number of checks on the input data, including a GRCh37/CGCh38 assembly check via the Ensembl REST API (Fig. 1B). Locally installed VEP is called for each coordinate which returns a list of transcripts which are then paired with associated isoforms. Also returned for each transcript are ENST, ENSG, HGVS identifiers (den Dunnen *et al.*, 2016), amino acid change, protein position, PolyPhen/SIFT score and VEP consequence. The transcript RefSeqs are retrieved from Ensembl BioMart. The UniProt canonical isoform is identified from the SWISS-PROT database. The amino acid identity at the position returned by VEP for the canonical isoform is checked against the corresponding position in the SWISS-PROT sequence. The RefSeq Select accession for each gene is retrieved from HGNC. The allele frequency of each variant in the natural population is retrieved from gnomAD. The amino acid conservation is calculated using the ScoreCons algorithm, while known disease associations for the amino acid position are retrieved from UniProt and ClinVar. CATH and Pfam domain memberships are also returned.

The UniProt canonical isoform sequence is searched against all PDBe sequences using FASTA. The alignments provide the mapping of the variant amino acid to its equivalent position in each 3D structure. The PDB accession code, chain, position and amino acid identity of the closest structure (according to alignment *E*-value) are provided, together with its resolution and sequence alignment quality. From this, and all other structure matches, information is taken about the variant residue’s context: whether it is a catalytic residue, or involved in a disulphide bond, or makes contact with DNA, protein, ligands or metals from PDBsum. This information is provided in the downloadable tab-separated file only. Output to screen includes the transcripts relating to the UniProt canonical isoform, protein position, colour-coded CADD score (Rentzsch *et al.*, 2019) and PDB structure. When a position cannot be mapped to the canonical isoform, clicking ‘more info’ displays a table of all transcripts with further information. All additional annotations are included in the downloadable file. A more detailed description of these methods can be found in the Supplementary Material and on the VarMap website: https://www.ebi.ac.uk/thornton-srv/databases/VarMap.

## 3 VarMap web tool

We present here the web tool ‘VarMap’ that automates the mapping of a list of single nucleotide polymorphisms (SNPs) to their corresponding UniProt canonical isoform sequence positions [via VEP (McLaren *et al.*, 2016) and SWISS-PROT (Boutet *et al.*, 2007)] and their position in the closest 3D structure in PDBe (wwPDB consortium, 2019). In addition to a screen output VarMap provides a downloadable tab-separated file containing additional annotations at the DNA sequence, protein sequence and protein structure levels extracted from various resources to help explain the role and interchangeability of each variant. When a position cannot be mapped to the canonical isoform, alternative information is provided for other transcripts.

Figure 1D–G shows how VarMap annotations can be used to analyze large datasets using ClinVar as an example. Figure 1D shows the proportion of variants that are SNPs, and of these the proportions that are coding. Figure 1E shows the variant types and Figure 1F shows that using homologous structures increases the proportion of variants that can be mapped to structure from 18 to 58%. Figure 1G demonstrates the wealth of information that can be extracted by considering all closely related structures. Tools that map only onto a single structure—and, furthermore, those that only perform the mapping if the protein structure is human—may lose this interaction data.

## 4 Discussion

In principle, the information provided by VarMap could be obtained manually using the following existing tools and databases: Ensembl (Cunningham *et al.*, 2019), VEP (McLaren *et al.*, 2016), UniProt (UniProt, 2019), SWISS-PROT (Boutet *et al.*, 2007), BioMart (Kinsella *et al.*, 2011), HGNC (Braschi *et al.*, 2019), CATH (Dawson *et al.*, 2017), Pfam (El-Gebali *et al.*, 2019), M-CSA (Ribeiro *et al.*, 2018), FASTA (Pearson, 2014), PDBsum (Laskowski *et al.*, 2018), ScoreCons (Valdar, 2002), gnomAD (Lek *et al.*, 2016) and ClinVar (Landrum *et al.*, 2018). However, this process would be prohibitively time-consuming for large datasets.

Tools exist that are similar to parts of VarMap, such as VAI (Hinrichs *et al.*, 2016), varQ (Radusky *et al.*, 2018), G23D (Solomon *et al.*, 2016), StructMAn (Gress *et al.*, 2016), mutfunc (Wagih *et al.*, 2018) and Decipher (Firth *et al.*, 2009), but they do not address transcript and isoform mapping to the same degree, or provide the same breadth of structural annotations. VarMap has several additional features compared to existing tools which makes it especially useful for the analysis of large datasets.

Firstly, the batch upload facility allows thousands of variants to be annotated concurrently. The preservation of input ID means that input lines can be directly cross-referenced with output lines. Secondly, VarMap partially annotates all transcript-isoform pairs, which may be important if variants are in non-reference transcripts or non-canonical isoforms. It also highlights whether the UniProt canonical isoform relates to the RefSeq select transcript. Thirdly, VarMap returns information on three aspects of each variant:

1. At the DNA level, consequences and pathogenicity scores are returned as well as the allele frequency of natural/disease associated variants at that position.
2. At the protein sequence level, conservation is calculated, membership of Pfam and CATH, and whether the residue represents a known catalytic site. Known disease associations with the affected amino acids are reported.
3. At the protein structure level, the representative structure with position and resolution is returned as are intermolecular interactions from homologous structures between the variant amino acid and ligands, proteins, nucleic acids and metals.

## 5 Conclusion

VarMap provides a wide range of annotations for single variants or any size genomic coordinate variant datasets. It is envisaged that it will be useful for clinical geneticists with patient variant data and researchers who wish to consider the environmental context and protein spatial distribution of genetic variants on structures. The data-rich, tab-separated output file facilitates intuitive sorting and filtering using simple parsing commands or spreadsheets, which require no expert knowledge of bioinformatics or structural biology.

## Funding

This work was supported by an EMBL-EBI/Sanger postdoctoral (ESPOD) fellowship (to J.D.S.).

*Conflict of Interest*: none declared.

## Supplementary Material

btz482_Supplementary_Data
