# Supplementary material for: VarMap: a web tool for mapping genomic coordinates to protein sequence and structure and retrieving protein structural annotations
Source: Bioinformatics. 2019 Jun 13;35(22):4854–6. doi: 10.1093/bioinformatics/btz482 (PMC6853667; doi:10.1093/bioinformatics/btz482)
Supplement: btz482_Supplementary_Data [file btz482_supplementary_data.pdf]

VarMap Supplementary Information

Method

Upload file
Users can upload a file of genomic coordinates to be analyzed by VarMap via the "Upload" button. The file should be a tab-separated file in either 4- or 5-column format (the latter corresponding to .vcf format):

4-column format

Chromosome	Coords	Reference base	Variant base
1	1014042	G	A

5-column (.vcf) format

Chromosome	Coords	Identifier	Reference base	Variant base
1	1014042	rs143888043	G	A

Chromosome: The chromosome number (1-22, X, Y, or MT).
Coords: DNA coordinates of the base – must be numeric.
Identifier: An optional identifier (e.g. RS number), which is ignored by the processing, but appears in the output for reference.
Reference base: The DNA base (A, C, G, or T) at the given coordinate position. If a sequence of bases is entered (e.g. corresponding to a deletion), VarMap will return the VEP results, but will not attempt to map onto a protein sequence or 3D structure.
Variant base: The DNA variant of interest (A, C, G, or T). Again, if a sequence of bases is entered (e.g. corresponding to an insertion), VarMap will return the VEP results, but will not attempt to map onto a protein sequence or 3D structure.

The uploaded file can contain additional tab-separated columns, but these will be ignored by VarMap. A heading line in the input file is optional and will also be ignored. As the upload of very large files can take a long time – and possibly result in the server timing out – it is recommended that the file be stripped of surplus data columns prior to upload. The maximum number of coordinates recommended is 50,000.

Selection and checking of build
Users can specify which genome build their DNA coordinates are taken from – either GRCh37 or GRCh38 – by clicking the appropriate radio button on the input form. If there are enough coordinates in the file (i.e. at least 20), VarMap will check the build automatically. It does this by taking a random set of 20 coordinates from the input file and checking the original base against that returned by the Ensemble REST API(Cunningham et al., 2019) for builds GRCh37 and GRCh38. The build giving the best agreement is then used for the entire set of coordinates in the file. Any coordinates then found not to match this build are flagged with a warning.

VEP
The first step in the process is to call VEP(McLaren et al., 2016). If the input contains fewer than 20 entries, the VEP API is called for each coordinate in turn, and progress is reported on screen. For larger data sets, the user is asked for their e-mail address so that the processing can be performed in batch mode on our processor farm using an in-house installation of VEP. A link to the results is then e-mailed to the user when all processing is complete.

VEP output
For each input coordinate, VEP returns the corresponding list of transcripts, identified by an ENST code, together with additional data (see table below). VarMap identifies the protein isoform corresponding to each ENST transcript using a list of transcript-isoform pairs (where the translated transcript sequence is identical to an isoform sequence) provided by

UniProt(UniProt, 2019). Given the isoform, the following data can be obtained: UniProt isoform accession number, amino acid position, amino acid change, gene symbol, PolyPhen(Adzhubei et al., 2013) score, SIFT (Vaser et al., 2016)score, CADD score(Rentzsch et al., 2019) and VEP consequence. Additionally, the RefSeq(O'Leary et al., 2016) accession for each transcript, where available, is retrieved via the Ensemble BioMart (Kinsella et al., 2011) download.

UniProtKB/SWISS-PROT canonical isoform

Of particular interest are isoforms that correspond to the canonical sequences in UniProtKB/SWISS-PROT(Boutet et al., 2007), as these will have the curated annotations. Other transcripts may map to alternative isoforms, or may not map to any isoform at all (i.e. the DNA variant is not in a known coding region of the genome). Only those mapping to the UniProtKB/SWISS-PROT canonical sequences are further annotated with 3D protein structural information (if available). The canonical is found by comparing a UniProt transcript-isoform pair list with SWISS-PROT.

VEP consequences

There is a wide range of possible consequences of any given variant, as defined by VEP. Those of interest here are missense, synonymous, stop gain, or stop loss variants. Others are likely to fall in an untranslated region such as a 5'UTR or intron, and no further mapping can take place for these. For missense and synonymous variants, the corresponding amino acid returned by VEP is checked against the amino acid at the given protein position in the SWISS-PROT sequence. A mismatch suggests a problem with the identification of the canonical isoform, so a warning is returned in the output.

VarMap output

The complete list of fields returned by VarMap for each valid variant is given in Table S1. The sources of additional data are:

- *RefSeq identifier* is retrieved from the HGNC database(Braschi et al., 2019) using the ENSG gene identifier returned by VEP. This is the Select RefSeq for the gene to which the variant maps, and may represent a transcript which is not associated with the UniProt canonical isoform.
- *Residue conservation* is computed using the ScoreCons algorithm(Valdar, 2002) from the pairwise alignments obtained from a BLAST(Pearson, 2014)search of the canonical protein sequence against the UniProt Knowledgebase.
- *Natural variants* are obtained from the gnomAD database(Lek et al., 2016) processed by VarMap to map DNA coordinates to protein residue positions.
- *Diseases* associated with variants at the given residue position come from UniProt and ClinVar(Landrum et al., 2018).
- *PFAM domain data* come from the PFAM(El-Gebali et al., 2019) ftp server.
- *CATH domain data* come from the CATH (Dawson et al., 2017) ftp server.
- *The closest PDB structure* is found by a FASTA(Pearson, 2014) search of the canonical protein sequence against the protein sequences in PDBe(ww, 2019). The closest match, by *E*-value, to the region of the protein covering the residue position of interest is taken. Other sufficiently close hits (i.e. sequence identity at least 30%) are retained for the structural information they can provide. For example, the closest PDB structure might comprise just the protein, whereas the structures of other, related proteins may have information about any intermolecular interactions the residue of interest might be involved in – e.g. interaction with ligand, DNA, metal, or other protein.
- *Secondary structure information* comes from PDBsum(Laskowski et al., 2018)and includes the secondary structure assignment (strand, helix or coil), whether the residue is a catalytic residue (as defined in M-CSA(Ribeiro et al., 2018), and whether it is part of a disulphide bond. Other information supplied by PDBsum includes any interactions the residue is involved in (i.e. with ligand, DNA, metal, or other protein).

The flow diagram below (figure S1) illustrates the VarMap pipeline and the sources of data it makes use of.

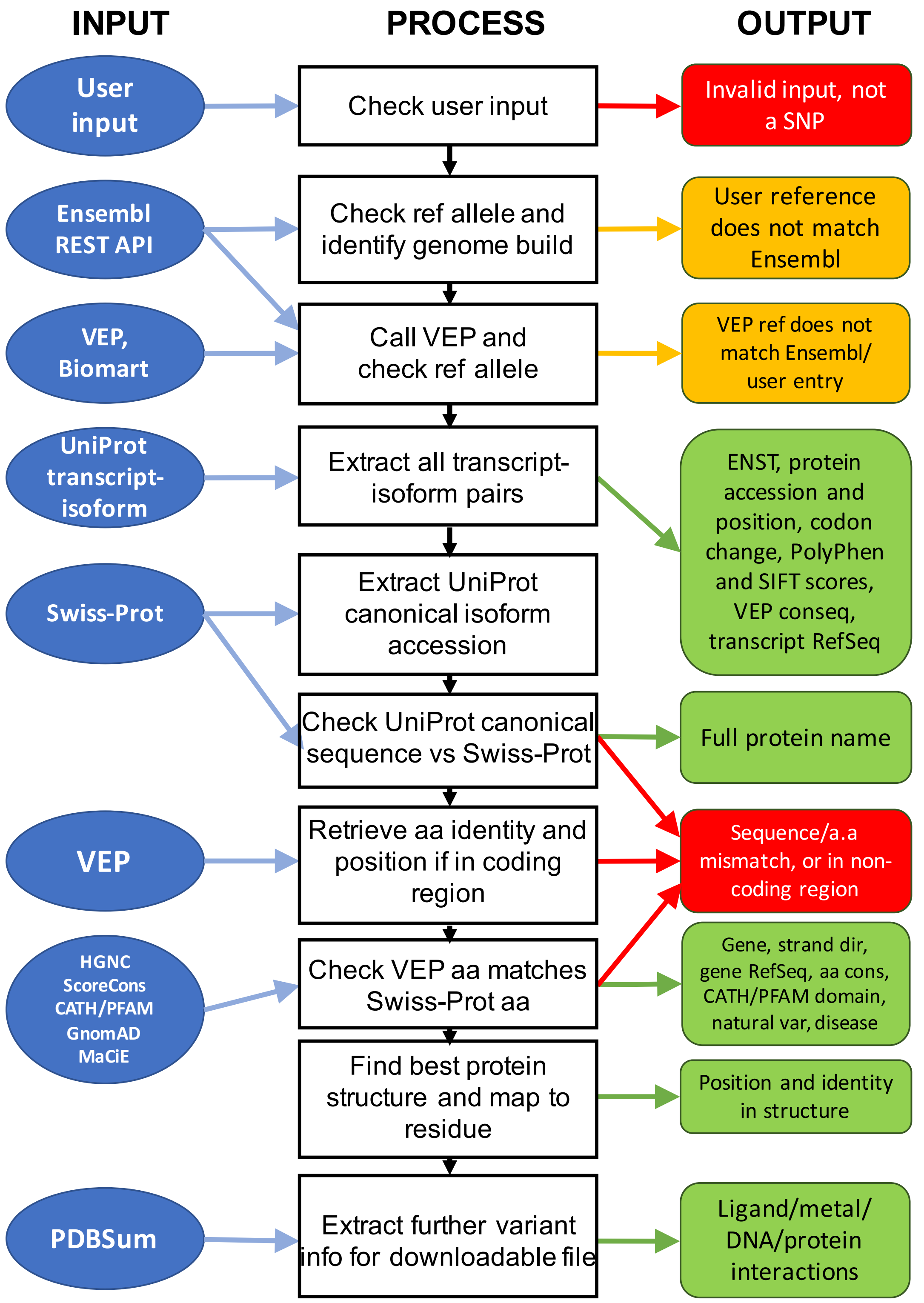

Figure S1. Schema for mapping from variant genomic coordinates to protein sequence. The blue left-most boxes show inputs from the user or databases. The middle column shows processes performed by the tool. The rightmost column shows the tool outputs with red indicating a critical error which prevents mapping, orange represents a tolerated error where mapping can still occur and green indicates successful annotation or mapping output.

**CHROMOSOME**	User entry	Must be 1-22 or X, Y, Mt (case insensitive)	If the chromosome is invalid the line is not run
**COORDS**	User entry	Must only contain numeric characters	If the coordinate is invalid the line is not run
**USER_BASE**	User entry	Must be a single base: A, G, C, or T. Sequences of bases will not be annotated	If the user base does not match Ensembl REST API a warning is returned but the line is still run
**USER_VARIANT**	User entry	Must be a single base: A, G, C, or T. Sequences of bases will not be annotated	Cannot be the same as the reference base as VEP returns nothing
**ENSEMBL_BASE**	Ensembl API	Retrieved from the relevant genome build (GRCh37 or GRCh38)	Compared with the user-entered base, and flagged if different
**VEP_CODING_BASE**	VEP	The reference base at this position in VEP	Compared with the user-entered base and Ensembl base, and flagged if different
**GENE**	Ensembl API	The standard Ensembl gene identifier	
**GENE_ACC**	VEP	Ensembl ENSG gene identifier for the transcript corresponding to the canonical isoform	
**REFSEQ_GENE_ACC**	HGNC	RefSeq accession for the gene	This RefSeq may not be for the transcript corresponding to the canonical isoform
**TRANSCRIPT**	VEP	All ENST transcript identifiers which map to the UniProt canonical isoform	Separated by “;”
**REFSEQ_TRANSCRIPT**	Ensembl BioMart	RefSeq for each transcript which corresponds to the canonical isoform if available	
**HGVS_C**	VEP	HGVS coding sequence name	
**HGVS_P**	VEP	HGVS protein sequence name	

**STRAND_DIR**	VEP	The strand direction for the gene (positive or negative)	For negative strand genes, the VEP_CODING_BASE will be the complement of the USER_BASE
**CODON_CHANGE**	VEP	Original codon and variant codon, with changed base shown in capitals (e.g. aGc/aAc)	
**VEP_AA**	VEP	Reference amino acid containing the variant base	
**UNIPROT_AA**	UniProt	Amino acid at the variant position in the canonical amino acid sequence	Amino acid identity checked against VEP_AA. Error returned on mismatch
**AA_CHANGE**	VEP	The amino acid change caused by the variant	Synonymous substitutions (ie no amino acid change) denoted by a "*"
**POLYPHEN_SCORE**	VEP	The probability that the amino acid substitution is damaging	
**SIFT_SCORE**	VEP	Prediction whether the amino acid change affects protein function	
**CADD_PHRED**	VEP/CADD	scaled variant rank relative to all possible substitutions	
**CADD_RAW**	VEP/CADD	The higher the score the more the variant is predicted to be deleterious	
**CADD_MARK**	VEP/CADD	A flag for internal use	
**UNIPROT_ACCESSION**	UniProt	UniProt accession code of the protein from the canonical isoform	
**PROTEIN_NAME**	UniProt	Full standard protein name	
**SEQ_NO**	VEP	The amino acid position in the UniProt canonical isoform sequence	All UniProt annotations are based on the numbering of the canonical isoform
**CHANGE_TYPE**	VEP	VEP consequence of the variant	
**ALL_TRANSCRIPTS**	VEP	A list of all transcript-isoform pairs separated by "/". Each gives: ENST, RefSeq, UniProt accession, amino acid position, amino acid change	If the data is unavailable, or not relevant, then a "-" is inserted

		(as X/Y), gene symbol, PolyPhen score, SIFT score, VEP consequence	
**NOTE**	VEP/ UniProt /Ensembl	Errors and warnings, such as mismatched reference alleles, which do not stop the mapping	
**GNOMAD_ALLELE_FREQUENCY**	VEP	Allele frequency of the variant from gnomAD.	
**NEGATIVE**		A flag (TRUE/FALSE) indicating whether the strand direction is negative	
**USER_ID**	(User)	Displays the user ID as provided in the third column of the input.	If no user ID is entered, an ID is generated according to the position in the input
**SYNONYMOUS**		A flag (TRUE/FALSE) indicating whether the amino acid change is a synonymous one	
**HAVE_PDB**		A flag (TRUE/FALSE) indicating whether the variant has been mapped onto a 3D protein structure in the PDB	The closest PDB structure, in terms of sequence identity and E-value, is selected for the region of sequence containing the variant
**PDB_UNIPROT_MATCH**		A flag (TRUE/FALSE) indicating whether the PDB structure is of the correct protein (as given by the UniProt accession)	A FALSE flag indicates that the closest 3D structure is from a different protein. The match stats are given below
**CLOSEST_PDB_CODE**	PDB	PDB code of the closest 3D structure in the PDB	
**PDB_CHAIN**	PDB	Corresponding PDB chain identifier	
**PDB_PROTEIN_NAME**	PDB	Protein name of the closest PDB structure	
**PDB_EXPT_TYPE**	PDB	Experimental method by which the structure was solved	X-RAY, NMR, etc.
**PDB_RESOLUTION**	PDB	X-ray resolution, in Ångstroms	
**PDB_RFACT**	PDB	R-factor from X-ray structure refinement	
**PDB_UNIPROT_ACC**	PDB	UniProt sequence corresponding to the 3D structure	

**PDB_IDENTITY**	FASTA	Sequence identity between the UniProt sequence (UNIPROT_ACCESSION) and the PDB sequence in (CLOSEST_PDB_CODE)	
**PDB_SW_SCORE**	FASTA	Smith-Waterman for the above match	
**PDB_E_VALUE**	FASTA	E-vale for the above match (cut-off is 0.001)	
**RES_NAME**	PDB	3-character residue name in PDB structure	
**RES_NUM**	PDB	Residue number in PDB structure	
**SST**	PDBsum	Secondary structure assignment (H=helix, E=strand, -=coil)	
**CAT_RES**	PDBsum	A flag (TRUE/FALSE) indicating whether the residue is a catalytic residue, as defined in MACiE	
**DISULPHIDE**	PDBsum	A flag (TRUE/FALSE) indicating whether the residue is disulphide-bonded cysteine	
**NTO_DNA**	PDBsum	Number of related 3D structures in which residue contacts DNA	
**NTO_LIGAND**	PDBsum	Number of related 3D structures in which residue contacts a bound ligand	
**NTO_METAL**	PDBsum	Number of related 3D structures in which residue contacts a bound metal ion	
**NTO_PROTEIN**	PDBsum	Number of related 3D structures in which residue is involved in a protein-protein interaction	
**NPDB_RES**	PDBsum	Number of related 3D structures that this residue can be mapped to	
**LIGANDS**	PDBsum	List of ligands in related 3D structures that the residue interacts with	
**METALS**	PDBsum	List of metal ions in related 3D structures that the residue interacts with	
**PFAM_DOMAIN**	PFAM	Pfam domain in which the residue is located	Identified by Pfam id

**PFAM_NAME**	PFAM	Name of the Pfam domain	
**CATH_DOMAIN**	CATH	CATH domain in which the residue is located	Identified by CATH id
**CATH_NAME**	CATH	Name of the CATH domain	
**RES_CONSERVATION**	ScoreCons	Residue conservation score (0.0-1.0)	Obtained from a BLAST search of the sequence against UniProt, a subsequent multiple sequence alignment and ScoreCons calculation
**NCONS_SEQS**	ScoreCons	Number of sequences used for calculating RES_CONSERVATION	
**DISEASES**	UniProt/ClinVar	List of diseases associated with the given variant	Identified by UniProt disease id
**DISEASE_VARIANTS**	UniProt/ClinVar	Numbers of associations for given variant	Separated by semi-colons, the counts are for: diseases; disease notes; mutagenesis experiments; DDD; ClinVar data
**NVARIANTS**	UniProt/ClinVar	Total number of variants in DISEASE_VARIANTS	
**NAT_VARIANTS**	VEP (gnomAD)	List of natural variants at this residue position	

Table S1. A description of each of the columns in the downloadable tab separated output file including the data source and additional information including error handling.

Method for Figure 1

Figure 1B - The ENSG gene identifier was extracted from the VarMap downloadable output. This was used to query the HGNC database for the RefSeq Select transcript identifier. The ENST identifiers corresponding to the transcripts which translate directly to the UniProt canonical isoform sequence were extracted from the VarMap output file. Ensembl Biomart was then queried using each ENST to retrieve all RefSeq identifiers associated with each. The transcript level Refseq identifiers were then cross referenced with the gene level RefSeq Select identifiers. If one of the transcript RefSeq identifiers matched the Select RefSeq then the variant was scored as a match – meaning that the Select RefSeq transcript translates directly to the Uniprot canonical isoform sequence. If no transcript RefSeq identifiers matched the Select RefSeq then this was scored as a mis-match – where the RefSeq transcript does not directly translate to the Uniprot canonical isoform. Variants were only considered for scoring if they could be mapped to the canonical isoform and if both transcript and gene level (select) RefSeq identifiers were present.

Figure 1D - The VarMap output columns USER_BASE and USER_VARIANT were used to count those variants were both the reference and variant allele were a single nucleotide.

Figure 1E - The VarMap output column CHANGE_TYPE was used to count the types of variant consequence and those that could not be mapped using the tool.

Figure 1F - The VarMap output column PDB_UNIPROT_MATCH was used to establish whether a variant could be mapped to the exact human protein structure corresponding to the gene or whether it could be mapped to a closely related homologous structure.

Figure 1G - The VarMap output columns NTO_DNA, NTO_LIGAND, NTO_METAL and NTO_PROTEIN were used to count how many variant amino acids have intermolecular contacts. If more than one contact is seen for a variant then both interactions are counted. If no interactions are seen then it is counted as 'no intermolecular interactions'.

Adzhubei, I. et al. (2013) Predicting functional effect of human missense mutations using PolyPhen-2. *Curr Protoc Hum Genet*, Chapter 7:Unit7 20.

Boutet, E. et al. (2007) UniProtKB/Swiss-Prot. *Methods Mol Biol*, 406:89-112.

Braschi, B. et al. (2019) Genenames.org: the HGNC and VGNC resources in 2019. *Nucleic Acids Res*, 47(D1):D786-D792.

Cunningham, F. et al. (2019) Ensembl 2019. *Nucleic Acids Res*, 47(D1):D745-D751.

Dawson, N.L. et al. (2017) CATH: an expanded resource to predict protein function through structure and sequence. *Nucleic Acids Res*, 45(D1):D289-D295.

El-Gebali, S. et al. (2019) The Pfam protein families database in 2019. *Nucleic Acids Res*, 47(D1):D427-D432.

Kinsella, R.J. et al. (2011) Ensembl BioMarts: a hub for data retrieval across taxonomic space. *Database (Oxford)*, 2011:bar030.

Landrum, M.J. et al. (2018) ClinVar: improving access to variant interpretations and supporting evidence. *Nucleic Acids Res*, 46(D1):D1062-D1067.

Laskowski, R.A. et al. (2018) PDBsum: Structural summaries of PDB entries. *Protein Sci*, 27(1):129-134.

Lek, M. et al. (2016) Analysis of protein-coding genetic variation in 60,706 humans. *Nature*, 536(7616):285-291.

McLaren, W. et al. (2016) The Ensembl Variant Effect Predictor. *Genome Biol*, 17(1):122.

O'Leary, N.A. et al. (2016) Reference sequence (RefSeq) database at NCBI: current status, taxonomic expansion, and functional annotation. *Nucleic Acids Res*, 44(D1):D733-745.

Pearson, W.R. (2014) BLAST and FASTA similarity searching for multiple sequence alignment. *Methods Mol Biol*, 1079:75-101.

Rentzsch, P. et al. (2019) CADD: predicting the deleteriousness of variants throughout the human genome. *Nucleic Acids Res*, 47(D1):D886-D894.

Ribeiro, A.J.M. et al. (2018) Mechanism and Catalytic Site Atlas (M-CSA): a database of enzyme reaction mechanisms and active sites. *Nucleic Acids Res*, 46(D1):D618-D623.

UniProt, C. (2019) UniProt: a worldwide hub of protein knowledge. *Nucleic Acids Res*, 47(D1):D506-D515.

Valdar, W.S. (2002) Scoring residue conservation. *Proteins*, 48(2):227-241.

Vaser, R. et al. (2016) SIFT missense predictions for genomes. *Nat Protoc*, 11(1):1-9.

ww, P.D.B.c. (2019) Protein Data Bank: the single global archive for 3D macromolecular structure data. *Nucleic Acids Res*, 47(D1):D520-D528.
